# Editorial: Retroconstruction of porous crystalline networks for a sustainable future

**DOI:** 10.3389/fchem.2025.1722377

**Published:** 2025-11-07

**Authors:** Xing Li

**Affiliations:** 1 Department of Chemistry, City University of Hong Kong, Kowloon, Hong Kong SAR, China; 2 Hong Kong Institute for Clean Energy, City University of Hong Kong, Kowloon, Hong Kong SAR, China

**Keywords:** porous crystalline networks, metal-organic frameworks (MOFs), covalent organic frameworks (COFs), hydrogen-bonded organic frameworks (HOFs), carbon capture, wastewater treatment, lithium-ion-coupled electron transfer, crystal engineering

The 2025 Nobel Prize in Chemistry, awarded to Susumu Kitagawa, Richard Robson, and Omar M. Yaghi, celebrates their pioneering work on metal-organic frameworks (MOFs) (Hoskins and Robson, 1990; Kondo et al., 1997; Yaghi et al., 1995), a class of porous crystalline networks (PCNs) built from metal nodes and organic linkers. This breakthrough introduced a new paradigm in materials design, enabling the creation of nanosized cavities with molecular precision. From this foundation, “reticular chemistry” (Yaghi et al., 2019) has flourished, expanding PCNs to include covalent organic frameworks (COFs) (Côté et al., 2005) and hydrogen-bonded organic frameworks (HOFs) (He et al., 2011). By linking molecular building blocks through strong bonds like coordination, covalent, or hydrogen interactions, reticular chemistry unlocks vast chemical diversity. This high designability encodes functions such as gas storage, separation, and catalysis into PCNs. Yet, despite these advances, industrial-scale “killer applications” remain scarce, with only a few showing real-world promise. With the groundwork laid, what lies ahead for PCNs?

Timed to coincide with the Nobel recognition, this Research Topic assembles five articles that bridge fundamental PCN research with sustainability challenges amid the climate crisis. The Research Topic spans structural modulation (Johnson et al.), materials hybridization (Hossain et al.), and applications in carbon capture (Cammarere et al.), energy storage (Ghuffar and Noh), and wastewater treatment (Mohammed Yaseen et al.). Central to this is our proposed “retroconstruction” approach, which merges retrosynthesis (designing molecules backward from desired outcomes) with retroengineering (deconstructing problems to identify solutions). Retroconstruction involves three steps: (1) identifying essential properties needed for real-world problems; (2) selecting molecular motifs, topologies, and pore structures to achieve them; and (3) developing efficient synthesis and processing methods. This framework shifts PCNs from serendipitous discoveries to targeted tools for sustainability.

Carbon capture is pivotal for curbing anthropogenic emissions and meeting the Paris Agreement’s goal of limiting global warming to 2 °C above preindustrial levels (Baker et al., 2018; Masson-Delmotte et al., 2018). Traditional amine scrubbing is energy-intensive, prone to sorbent degradation, and challenging to retrofit (Rochelle Gary, 2009). PCNs, with their tunable porosity for reversible gas adsorption, offer a solvent-free alternative (Lin et al., 2021; Zhou et al., 2024). However, water vapor in flue gases or air often competes with CO_2_, reducing efficacy (Siegelman et al., 2019). Cammarere et al. review water-enhanced CO_2_ capture in MOFs, highlighting mechanisms like dipole-quadrupole interactions, water dissociation creating new adsorption sites, nanopocket confinement, and chemical sorption via carbamates, carbamic acids, or bicarbonates. By retroconstructing MOFs by choosing specific metal nodes, ligands, topologies, and pores, these designs optimize performance under humid conditions, paving the way for efficient point-source or direct air capture.

Equally vital for carbon neutrality are renewable energy technologies, which demand advanced electrochemical devices like batteries and electrolyzers. A key hurdle is understanding lithium-ion-coupled electron transfer (LCET) reactions at electrode-electrolyte interfaces, including their thermodynamics and Li^+^-electron stoichiometry, which is vital in cathode design in Li-ion batteries (Nikitina et al., 2017). Bulk metal oxides often fail to show Nernstian behavior with Li^+^ concentrations, precluding the derivation of LCET thermochemistry. Ghuffar and Noh address this through retroconstruction: a Zr-based MOF confines tungsten oxide (WO_x_) into nanoparticles within its pores, creating an ideal platform for LCET studies. This nano-confinement reveals precise stoichiometry and Gibbs free energy, offering insights for energy storage and conversion that align with sustainable electrification.

Clean water access, another UN Sustainable Development Goal, faces threats from chemical pollutants and pathogens. Retroconstructing PCNs can yield multifunctional materials for wastewater treatment (Li et al., 2021). Mohammed Yaseen et al. introduce a vanadium-based MOF with 2,2′-bipyridine-4,4′-dicarboxylic acid ligands, combining porosity for dye adsorption with antimicrobial properties against agents like *E. coli*. This synergy derived from bioactive ligands and porosity demonstrates how targeted design addresses dual challenges in water purification.

Practical deployment of PCNs is often hampered by their powdery form, limiting processability. Hossain et al. review covalent integration of polymers with PCNs, such as MOFs, COFs and HOFs, to create hybrids with enhanced stability, flexibility, and scalability. These strategies overcome traditional drawbacks, enabling retroconstruction for industrial applications like membranes or coatings.

At the heart of PCN functionality are their topologies and porosities, yet MOF structures can be unpredictable due to metal multivalency and ligand conformations (Jiang et al., 2021). Johnson et al. probe this in tetraphenylethene-based MOFs, using rotamer and pillar ligands to control net dimensionality, pore sizes, and surface areas. This modular approach exemplifies retroconstruction, facilitating rational design for tailored applications without reinventing building blocks.

These articles collectively illustrate retroconstruction’s power: by deconstructing sustainability problems and reassembling PCNs accordingly, we can accelerate real-world impact. Challenges persist in scalability, cost, and environmental stability, which must be addressed through interdisciplinary efforts, including AI-driven design and lifecycle assessments. Nonetheless, the future of PCNs centers on rational innovation to transform energy systems, reduce emissions, and ensure resource equity, aligning with the UN’s Sustainable Development Goals.

In this Nobel-inspired moment, retroconstruction invites us to envision PCNs not as mere materials, but as architects of a sustainable future that is porous with possibility.
